# Experimental simulation of symmetry-protected higher-order exceptional points with single photons

**DOI:** 10.1126/sciadv.adi0732

**Published:** 2023-08-23

**Authors:** Kunkun Wang, Lei Xiao, Haiqing Lin, Wei Yi, Emil J. Bergholtz, Peng Xue

**Affiliations:** ^1^ School of Physics and Optoelectronic Engineering, Anhui University, Hefei 230601, China.; ^2^ Beijing Computational Science Research Center, Beijing 100084, China.; ^3^ School of Physics, Zhejiang University, Hangzhou 310030, China.; ^4^ Key Laboratory of Quantum Information, University of Science and Technology of China, CAS, Hefei 230026, China.; ^5^ CAS Center for Excellence in Quantum Information and Quantum Physics, University of Science and Technology of China, Hefei 230026, China.; ^6^ Department of Physics, Stockholm University, AlbaNova University Center, 106 91 Stockholm, Sweden.

## Abstract

Exceptional points (EPs) of non-Hermitian (NH) systems have recently attracted increasing attention due to their rich phenomenology and intriguing applications. Compared to the predominantly studied second-order EPs, higher-order EPs have been assumed to play a much less prominent role because they generically require the tuning of more parameters. Here, we experimentally simulate two-dimensional topological NH band structures using single-photon interferometry, and observe topologically stable third-order EPs obtained by tuning only two real parameters in the presence of symmetry. In particular, we explore how different symmetries stabilize qualitatively different third-order EPs: the parity-time symmetry leads to a generic cube-root dispersion, while a generalized chiral symmetry implies a square-root dispersion coexisting with a flat band. Additionally, we simulate fourfold degeneracies, composed of the non-defective twofold degeneracies and second-order EPs. Our work reveals the abundant and conceptually richer higher-order EPs protected by symmetries and offers a versatile platform for further research on topological NH systems.

## Teaser

Simulation with single photons reveals distinct classes of higher-order exceptional points protected by non-Hermitian symmetries.

## INTRODUCTION

Exceptional points (EPs) of non-Hermitian (NH) systems are branch point singularities in the parameter space, which emerge at the turning points of the dispersions of the interface states (*1*–*4*). EPs exhibit fascinating topological phenomena (*5*–*8*) and lead to intriguing applications, such as sensing (*9*–*15*), unidirectional wave propagation (*16*–*19*), chiral laser emission (*20*), laser linewidth broadening (*21*), and laser mode selection (*22*–*25*).

The simplest case of EPs is the second-order EP, that is, twofold degeneracy, which intuitively occurs in a two-dimensional (2D) system, but can be promoted to knotted exceptional lines in three dimensions (*26*–*29*). Generally, an *n*th-order EP is stable in a (2*n* − 2)–dimensional NH system (*30*), which makes them qualitatively more abundant than degeneracies in Hermitian systems. When exploring the NH systems with symmetries, the dimension for the occurrence of generic second-order EPs can be further reduced from two to one, leading to the observation of stable second-order EPs even in 1D systems (*31*–*34*). Similarly, generic NH symmetries have been found to reduce the dimension for the occurrence of the third-order EPs from four to two (*35*–*37*). Moreover, different symmetries may also entail qualitatively different phenomenology for EPs with the same order (*35*). Although higher-order EPs have been studied by a number of works theoretically, their experimental realization and direct observation appear to be rather difficult, as ingenious designs are required to simulate the NH dynamics (*38*, *39*) and explore the band structures.

Here, by using single-photon interferometry, we overcome the difficulty of building NH systems with a large number of tunable parameters and simulate a 2D NH system in the reciprocal space. With interferometric measurements, complex eigenenergies are measured directly, enabling us to construct the band structures of the 2D NH system. In particular, we experimentally observe and characterize two distinct types of symmetry-protected third-order EPs. We also experimentally confirm the stability of the third-order EPs with respect to perturbations. Because they are protected by either the parity-time (PT) or a generalized chiral P symmetry, these EPs disappear upon introducing symmetry-breaking perturbations. Our experimental results demonstrate that the energy near the third-order EP exhibits a generic ∼*k*^1/3^ dispersion enforced by PT symmetry. The coexistent exceptional ring (ER) composed of the second-order EP bounds an open Fermi surface, which is also the boundary between the PT-unbroken and PT-broken regimes. By contrast, an anomalous ∼*k*^1/2^ dispersion is observed away from the chiral P symmetry–protected third-order EPs.

We further extend our experiment to simulate 2D NH models with four bands, where PT and P symmetries have radically different implications. For the PT symmetry, the third-order EPs and ER are present regardless of the additional bands. In contrast, for the P-symmetry case, the third-order EPs are forbidden by the additional bands. We also observe fourfold degeneracies, each composed of a non-defective twofold degeneracy and a second-order EP (*40*, *41*). Our results thus experimentally expose the abundance of higher-order EPs whose codimensions are reduced in the presence of NH symmetries, thus offering potential applications in efficient device design.

## RESULTS

### Experimental setup

We explore the symmetry-protected third-order EPs by simulating the dynamics of the corresponding three-band NH Bloch Hamiltonians *H*(**k**). The complex eigenenergies are measured via interferometric measurements (*26*).

**Fig. 1.**
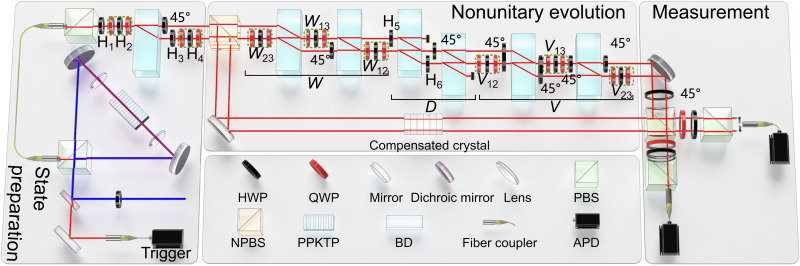
Experimental setup. State preparation is achieved by subjecting the signal photons to a polarizing beam splitter (PBS), five half–wave plates (HWPs), four quarter–wave plates (QWPs) and a beam displacer (BD). Single photons are generated by spontaneous parametric down-conversion (SPDC) in the periodically poled potassium titanyl phosphate (PPKTP) crystal. Two other spatial modes of photons are introduced after the photons pass through a non-polarizing beam splitter (NPBS). The transmitted photons experience a nonunitary evolution that is realized via the interferometric network. The interferometric network is composed of sets of wave plates and BDs. The reflected photons serve as references for interferometric measurements. The eigenenergies are encoded in the complex phase shift between the photons in different spatial modes, which are measured via interferometric measurements. The photons are detected by avalanche photodiodes (APDs).

As illustrated in Fig. 1, our experiment involves three stages: state preparation, nonunitary evolution, and measurement. The basis states of the three-band system are encoded into the hybrid polarization-spatial modes of single photons. In the preparation stage, the states of single photons are initialized in the eigenstate ψ*_j_*(**k**) of *H*(**k**) with the corresponding eigenenergy *E_j_*. After passing through a non-polarizing beam splitter (NPBS), the transmitted photons go through the nonunitary evolution governed by *H*(**k**) and the reflected photons remain unchanged. They interfere at the second polarizing beam splitter (PBS) for interferometric measurements. To circumvent the quantum limit on achieving gain for single photons in the nonunitary-evolution stage, we map the time- evolution operator *U* = *e*^−*iH*(**k**)τ^ to a dissipative one $\tilde{U}=U/\sqrt{\text{Λ}}$. Here, we fix the evolution time τ = 1 and ℏ = 1, and take Λ = max_*j*_∣ζ_*j*_∣, where ζ_*j*_ is the eigenvalue of *e*^−*iH*^*e*^*iH*^†^^ (*26*, *42*, *43*). The corresponding effective NH Hamiltonian for the mapped dissipative system is thus $\tilde{H}(k)=H(k)+i\ln\sqrt{1/\text{Λ}}\sigma_{0}$, where σ_0_ is a 3 × 3 identity matrix. It follows that $\tilde{H}(k)$ and *H*(**k**) have the same eigenstates, and their eigenenergies are related and satisfy ${\tilde{E}}_{j}=E_{j}-i\ln\sqrt{\text{Λ}}$. In the measurement stage, the complex inner products of $\xi =e^{-i{\tilde{E}}_{j}}=\;\langle \psi_{j}|\tilde{U}|\psi_{j}\rangle$ are obtained through the interferometric measurements. The corresponding eigenenergies are then calculated from the experimentally measured ξ (see Materials and Methods).

### PT symmetry–protected third-order EPs

First, we consider three-band linearized, higher-spin Dirac-like NH Hamiltonians with PT symmetry in a 2D reciprocal space (*35*).

$$H_{PT}=k_{x}\lambda_{1}+i\varepsilon (\lambda_{2}+\lambda_{4}+\lambda_{5})+(k_{y}-i\varepsilon )\lambda_{6}-(k_{y}+i\varepsilon )\lambda_{7}-\frac{\varepsilon }{2}(\lambda_{3}+\sqrt{3}\lambda_{8})$$ (1)

Here λ*_i_* (*i* = 1, 2, ⋯, 8) denotes the Gell-Mann matrix (*44*). The model is Hermitian at ɛ = 0. Non-Hermiticity is introduced through finite ɛ. Under the PT symmetry, $(PT)H_{PT}^{*}{\left(PT\right)}^{-1}=H_{PT}$ is satisfied, where *P* = diag (1, −1, 1) and *T* = diag (−1, 1, *i*) are unitary matrices associated with the parity and time-reversal operators (*45*), respectively, satisfying TT^*^ = ±1 and TP^*^ = *PT*.

**Fig. 2.**
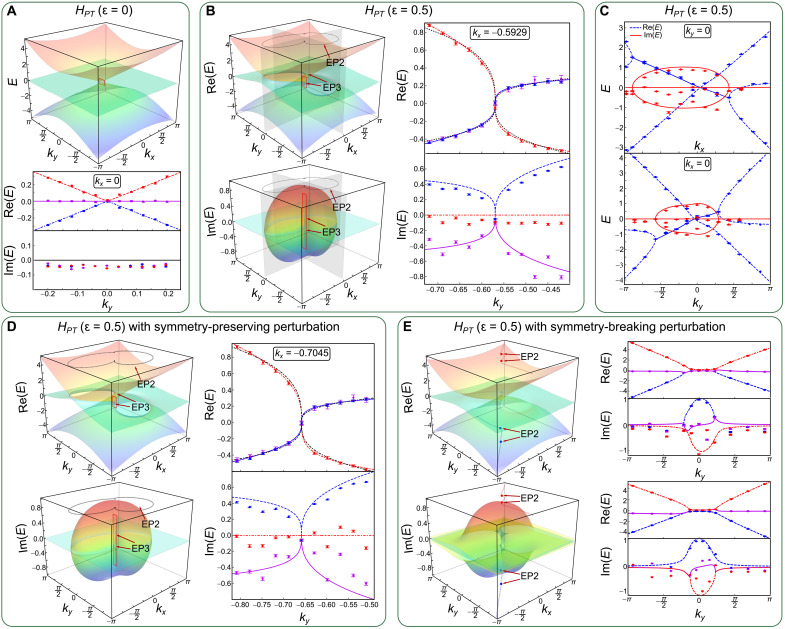
Observation of the PT symmetry–protected third-order EPs. The real and imaginary parts of the eigenenergies of *H_PT_* with ɛ = 0 (**A**) and ɛ = 0.5 (**B** and **C**), respectively, as functions of the momentum. The colored surfaces correspond to the theoretical results, where parameters for the experimental measurements are chosen within the range of red squares. Black dotted lines in the right columns of (B) and (D) correspond to the results fitted by ∼*k*^1/3^. The measured real (blue) and imaginary (red) parts of the eigenenergies with *k_y_* = 0 and *k_x_* = 0 as functions of *k_x_* and *k_y_* are shown in the top and bottom panels of (C). Effects due to the symmetry-preserving perturbation *i*π(λ_4_ + λ_5_)/20 and the symmetry-broken perturbation $\sum_{i=1}^{8}\delta_{i}\lambda_{i}$ for *H_PT_* with ɛ = 0.5 are shown in (**D** and **E**), respectively, where δ*_i_* ∈ [−π/20, π/20] are chosen randomly. The red and blue dotted lines in the left column of (E) depict the generalized arc degeneracies, where the measured results along them are shown in the right column. Experimental data are represented by symbols and theoretical results are denoted by colored lines. Error bars are obtained by assuming Poisson statistics in the photon-number fluctuations, indicating the statistical uncertainty. EP2, the second-order EP; EP3, the third-order EP.

Figure 2 presents the real and imaginary parts of the eigenenergies of *H_PT_*. At ɛ = 0, the Hamiltonian features a triple degeneracy, where two conical bands touch a flat band at *k_x_* = *k_y_* = 0. As shown in Fig. 2A, by fixing *k_x_* = 0, we experimentally observe a linear dispersion near the degenerate point, consistent with the theoretical prediction (*35*).

As illustrated in Fig. 2B, at ɛ = 0.5, the degenerate point splits into two third-order EPs, which occur at {*k_x_* = −0.5929, *k_y_* = −0.5694} and {*k_x_* = 0.4701, *k_y_* = 0.6241}, respectively. Focusing on one of the third-order EPs, we fix *k_x_* = −0.5929 and sample 11 different values of *k_y_*. The measured real and imaginary parts of the eigenenergies are shown in the right column of Fig. 2B. By fitting the power exponents with the formula ∼*k*^β^, we confirm that the real eigenenergy exhibits a generic cube-root dispersion (∼*k*^1/3^) near the third-order EP.

Furthermore, an ER emerges in the momentum space, which is particularly interesting as the ER signals the PT transitions and bounds an open Fermi surface (*35*). Because the ER is a collection of second-order EPs, we reveal its existence in Fig. 2C, where we measure the eigenenergies of *H_PT_* along the lines of *k_x_* = 0 and *k_y_* = 0, respectively. The second-order EPs are observed as the bifurcations of the real eigenenergies. In between the two EPs, the imaginary parts of all the eigenenergies are nonzero, indicating the PT symmetry–broken region. Therein, two of the measured eigenergies are approximately equal, indicating the emergence of an open Fermi surface.

As a feature of symmetry protection, the existence of the third-order EPs is robust against symmetry-preserving perturbations. As shown in Fig. 2D, under a small PT symmetry–preserving perturbation *i*π(λ_4_ + λ_5_)/20, both third-order EPs persist, but are shifted in parameter space. By contrast, the EPs disappear as illustrated in Fig. 2E, by introducing a general, symmetry-breaking perturbation $\sum_{i=1}^{8}\delta_{i}\lambda_{i}$ (δ*_i_* ∈ [−π/20, π/20]). The perturbed spectrum exhibits branch cuts that are terminated by paired second-order EPs (*41*). These paired second-order EPs further reveal a transition from regions where the real parts of the eigenenergies are degenerate, to those with degeneracy in the imaginary parts.

### P symmetry–protected third-order EPs

**Fig. 3.**
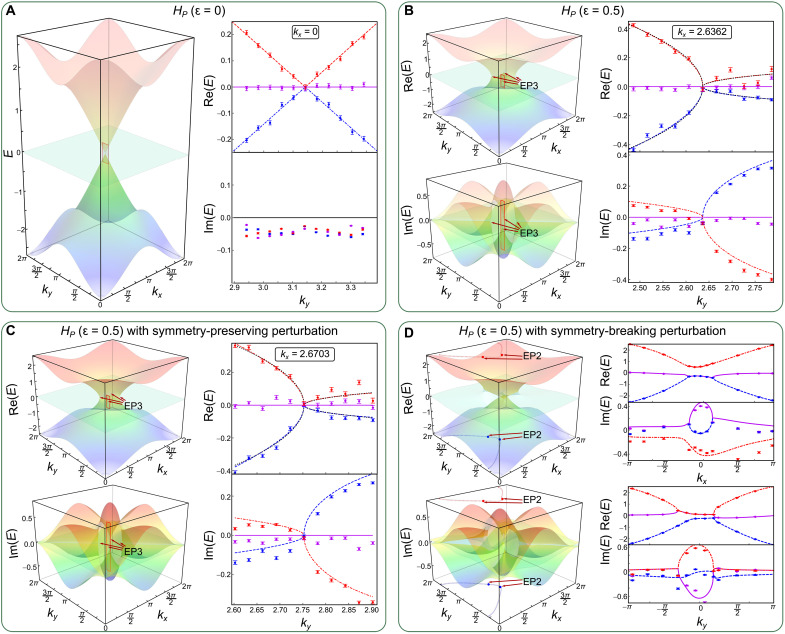
Observation of the P symmetry–protected third-order EPs. The real and imaginary parts of the eigenenergies of *H_P_* with ɛ = 0 (**A**) and ɛ = 0.5 (**B**), respectively, as functions of the momentum. The colored surfaces correspond to the theoretical results, where parameters for the experimental measurements are chosen within the range of red squares. Black dotted lines in the right columns of (B) and (C) correspond to the results fitted by ∼*k*^1/2^. Effects due to the symmetry-preserving perturbation *i*πλ_1_/20 and the symmetry-broken perturbation $\sum_{i=1}^{8}\delta_{i}\lambda_{i}$ for *H_P_* with ɛ = 0.5 are shown in (**C** and **D**), respectively, where δ*_i_* ∈ [−π/20, π/20] are chosen randomly. The red and blue dotted lines in the left column of (D) depict the generalized arc degeneracies, where the measured results along them are shown in the right column. Experimental data are represented by symbols and theoretical results by colored lines. Error bars are obtained by assuming Poisson statistics in the photon-number fluctuations, indicating the statistical uncertainty.

To demonstrate the features of NH model with P symmetry, we consider an NH Lieb lattice (*35*, *46*)

$$H_{P}=(1+\cos k_{x}-i\varepsilon )\lambda_{1}+(1+\cos k_{y}+i\varepsilon )\lambda_{6}-\sin k_{x}\lambda_{2}-\sin k_{y}\lambda_{7}$$ (2)

which satisfies the relation *H_P_* = −*PH_P_P*^−1^. Note that we consider the case where this symmetry acts locally in momentum space. For ɛ = 0, *H_P_* corresponds to the Hermitian Lieb lattice model. In the momentum space, similar to the case of *H_PT_*, a triple degeneracy of the eigenenergy exists at *k_x_* = *k_y_* = π, from which emerges two dispersive bands with linear scaling (see Fig. 3A). We introduce the NH term by setting ɛ = 0.5. The triple degeneracy then splits into four third-order EPs that locate at (*k_x_* = ±2.6362,  *k_y_* = ±2.6362). Focusing on one of the EPs, we fix *k_x_* = 2.6362 and sample 11 different *k_y_*. In Fig. 3B, we show the measured real and imaginary components of the eigenenergies, where the third-order EP is visible near *k_y_* = 2.6362. Different from the PT symmetry–protected case, the two nonzero real bands exhibit an anomalous square-root dispersion near the third-order EP, that is, ∼*k*^1/2^. This is a typical behavior associated with the second-order EPs. As shown in Fig. 3C, we introduce a P symmetry–preserving perturbation in the form of *i*πλ_1_/20. The four third-order EPs persist, with a small shift in the parameter space. Similar to *H_PT_*, third-order EPs are destroyed by the general, symmetry-breaking perturbation $\sum_{i=1}^{8}\delta_{i}\lambda_{i}$, leading to a branch cut terminated by a pair of second-order EPs (see Fig. 3D).

**Fig. 4.**
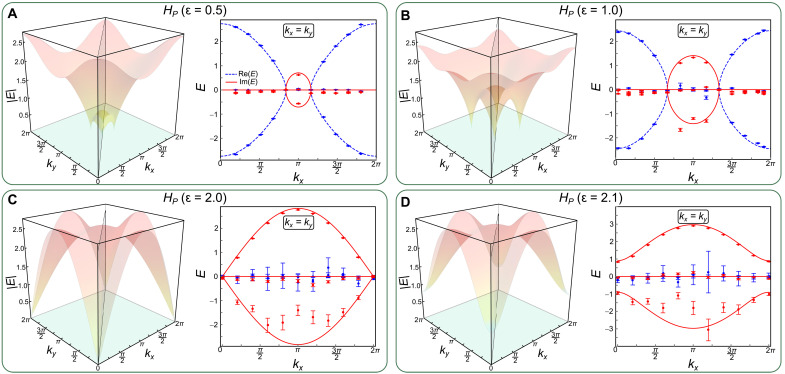
Energy spectra for the P-symmetric three-band model. The colored surfaces correspond to the absolute values for the eigenenergies of *H_P_* with ɛ = 0.5 (**A**), ɛ = 1.0 (**B**), ɛ = 2.0 (**C**), and ɛ = 2.1 (**D**), respectively. The gray planes correspond to the surfaces with *k_x_* = *k_y_*. Measured real (blue) and imaginary (red) results are represented by symbols and the corresponding theoretical ones by lines. Error bars are obtained by assuming Poisson statistics in the photon-number fluctuations, indicating the statistical uncertainty.

Accompanying the P symmetry–protected third-order EPs of *H_P_*, there are arc-like degeneracies along the lines of *k_x_* = ±*k_y_*, which correspond to the real and imaginary Fermi arcs with Re[*E*_±_] = 0 and Im[*E*_±_] = 0, respectively (*35*). Experimentally, we again sample 11 different parameters along the line of *k_x_* = *k_y_* for *H_P_* with ɛ = 0.5. In Fig. 4A, we show the measured real and imaginary components of the bands, where both the real and imaginary Fermi arcs can be observed. The boundaries between them give the locations of the third-order EPs. With increasing ɛ, as illustrated in Fig. 4 (B and C), the third-order EPs move from the center of the Brillouin zone near (*k_x_* = π, *k_y_* = π) to the edges. At ɛ = 2.0, the imaginary Fermi arcs give way to real Fermi arcs, as pairs of third-order EPs recombine, giving rise to linear dispersions at the EPs. Further increasing ɛ beyond 2, as shown in Fig. 4D with ɛ = 2.1, the third-order EPs completely disappear, as the spectrum opens up complex gaps. The real Fermi arcs develop into closed line degeneracies with Re[*E*_±_] = 0, slicing through the entire Brillouin zone.

### Generalization to symmetry-protected four-band models

We extend the system to the symmetry-protected four-band model governed by the Hamiltonian

$$\begin{array}{c} H_{PT}^{\prime }=k_{x}{\text{Γ}}_{1}+i\varepsilon ({\text{Γ}}_{2}+{\text{Γ}}_{4}+{\text{Γ}}_{5})+(k_{y}-i\varepsilon ){\text{Γ}}_{6}\\ -(k_{y}+i\varepsilon ){\text{Γ}}_{7}-\frac{\varepsilon }{2}({\text{Γ}}_{3}+\sqrt{3}{\text{Γ}}_{8})+\frac{{\text{Γ}}_{0}-\sqrt{6}{\text{Γ}}_{15}}{4} \end{array}$$ (3)

which is PT symmetric, with the symmetry operators *P*′ = diag (1, −1, 1, 1) and *T*′ = diag (−1, 1, *i*, 1). Here, Γ_0_ is a 4 × 4 identity matrix and Γ*_i_* (*i* = 1, 2, ⋯, 15) denote the Gell-Mann matrices that span the Lie algebra of the SU(4) group.

**Fig. 5.**
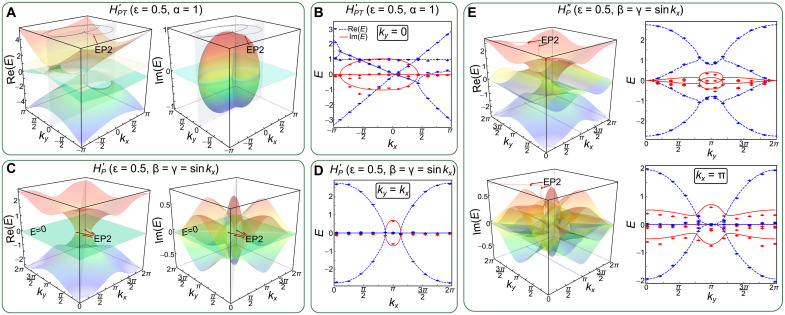
Energy spectra for four-band models. The colored surfaces correspond to the theoretical real and imaginary energy spectra of $H_{PT}^{\prime }$ with α = 1 (**A**), $H_{P}^{\prime }$ with β = γ = sin*k_x_* (**C**), and $H_{P}^{\prime \prime }$ with β = γ = sin*k_x_* (**E**) by setting ɛ = 0.5. The gray planes correspond to the surfaces with *k_y_* = 0 (A) and *k_y_* = *k_x_* (C), respectively, as chosen in our experiment. Measured real (blue) and imaginary (red) results are represented by points and the corresponding theoretical ones by lines for $H_{PT}^{\prime }$ (**B**) and for $H_{P}^{\prime }$ (**D**), respectively. The red dotted lines in the left column of (E) depict the generalized arc-like degeneracies. The blue dotted line corresponds to the non-defective twofold degenerate line with *k_x_* = π. The lines in the right column correspond to the theoretical results along the red dotted line (top) and *k_x_* = π (bottom), respectively. Measured results are represented by symbols. Error bars are obtained by assuming Poisson statistics in the photon-number fluctuations, indicating the statistical uncertainty.

Compared to *H_PT_* in Eq. 1, the eigenspectrum of $H_{PT}^{\prime }$ has an additional flat band at *E* = α. As shown in Fig. 5A, third-order EPs persist under α = 1 and ɛ = 0.5, at the same locations as those of *H_PT_*. A manifest difference is the emergence of an additional ER composed of the second-order EPs in the PT-symmetric four-band model. In Fig. 5B, by sampling the momentum of *k_x_* along the line of *k_y_* = 0, we experimentally confirm the existence of the extra real eigenenergy and the additional ER.

For P symmetry–protected four-band models, the restricted NH Hamiltonian becomes

$$\begin{array}{c} H_{P}^{\prime }=(1+\cos k_{x}-i\varepsilon ){\text{Γ}}_{1}+(1+\cos k_{y}+i\varepsilon ){\text{Γ}}_{6}-\sin k_{x}{\text{Γ}}_{2}-\sin k_{y}{\text{Γ}}_{7}\\ +\frac{\left(\beta +\gamma \right)}{2}{\text{Γ}}_{11}+\frac{i(\beta -\gamma )}{2}{\text{Γ}}_{12} \end{array}$$ (4)

where β and γ are arbitrary complex numbers. Here, Hamiltonian $H_{P}^{\prime }$ satisfies the relation $\det[H_{P}^{\prime }]=\mathrm{Tr}(H_{P}^{\prime })=\mathrm{Tr}(H_{P}^{\prime 3})=0$. Its corresponding eigenenergies are $\left\{0,0,E_{\pm }^{\prime }\right\}$ (*37*) with $E_{\pm }^{\prime }=\pm \sqrt{4+\beta \gamma -2\varepsilon^{2}+(2-2i\varepsilon )\cos k_{x}+(2+2i\varepsilon )\cos k_{y}}$, where the third-order EPs are destroyed by the additional band. The two degenerate flat bands at zero energy correspond to the non-defective degeneracies with two distinct eigenstates (*40*, *41*). Setting ɛ = 0.5 and tuning *k_x_* and *k_y_*, we confirm that the two dispersive bands $E_{\pm }^{\prime }$ touch each other at $E_{\pm }^{\prime }=0$, to form the second-order EPs, where the local degeneracies become fourfold (see Fig. 5, C and D).

Such fourfold degeneracies are not protected by the P symmetry. Changing the P-symmetry operator to *P*″ = diag (1, −1, 1, −1) (*35*), we choose the NH Hamiltonian in the form

$$\begin{array}{c} H_{P}^{\prime \prime }=(1+\cos k_{x}-i\varepsilon ){\text{Γ}}_{1}+(1+\cos k_{y}+i\varepsilon ){\text{Γ}}_{6}-\sin k_{x}{\text{Γ}}_{2}-\sin k_{y}{\text{Γ}}_{7}\\ +\frac{\left(\beta +\gamma \right)}{2}{\text{Γ}}_{13}+\frac{i(\beta -\gamma )}{2}{\text{Γ}}_{14} \end{array}$$ (5)

As shown in Fig. 5E, the fourfold degeneracies are gapped out, with β = γ = sin*k_x_* and ɛ = 0.5.

## DISCUSSION

By experimentally studying the symmetry-protected higher-order EPs of a series of 2D NH models, our work provides the first experimental confirmation that symmetries qualitatively enrich the phenomenology of higher-order degeneracies in the NH systems. By revealing the abundance of higher-order EPs whose codimensions are reduced in the presence of NH symmetries, our results have rich implications for efficient device design.

EPs are generic features of the effective description of a vast range of complex systems ranging from mechanical systems to strongly interacting quantum materials. In contrast, our simple experimental scheme is highly controllable and scalable, and can be readily extended to the detailed study of NH models with arbitrary design and physical origin. It thus constitutes a versatile and efficient platform for the systematic exploration of eigenspectrum and dynamical properties in NH settings.

## MATERIALS AND METHODS

### Initial state preparation

As illustrated in Fig. 1, our experimental setup can be used to prepare arbitrary pure qutrit states. The basis states of the three-band systems are encoded by the polarizations and spatial modes of single photons, with ∣0〉 ⇔ ∣*H*_1_〉, ∣1〉 ⇔ ∣*H*_2_〉, and ∣2〉 ⇔ ∣*V*_2_〉. Here, the subscripts denote the different spatial modes and *H* (*V*) denotes the horizontal (vertical) polarization of single photons. To prepare the generic qutrit state *a*∣*H*_1_〉 + *be*^*i*ϕ_1_^∣*H*_2_〉 + *ce*^*i*ϕ_2_^∣*V*_2_〉, the photons are first initialized to the horizontal polarization by passing them through a PBS. The two spatial modes of photons are introduced after the photons pass through a beam displacer. The real coefficients {*a*, *b*, *c*} with *a*^2^ + *b*^2^ + *c*^2^ = 1 can be adjusted by controlling half–wave plates (HWPs) with the setting angles of H_1_ = ( arcsin*a*)/2 and H_3_ = ( arctan*c*/*b*)/2. As for the relative phases {ϕ_1_, ϕ_2_} ranging from 0 to 2π, they can be adjusted by the sandwich-type set of HWP and quarter–wave plates (QWPs), denoted as QWP-HWP-QWP. Setting the angles of QWPs at 45° and HWP at 45° − 180°ϕ/π, one can achieve the phase operation of diag(*e*^2*i*ϕ^, *e*^−2*i*ϕ^). Thus, by setting H_2_ = 45°(ϕ_1_ + ϕ_2_)/π + 56.25° and H_4_ = 45°(ϕ_2_ − ϕ_1_) + 11.25°, we can tune the relative phases accordingly.

It follows that the initial state can be exactly prepared in one of the eigenstates of the NH Hamiltonian with prior information. Even if the eigenstates are unknown, we can still generate them as initial states by maximizing the probability of $P={|\langle \text{Ψ}|\tilde{U}|\text{Ψ}\rangle |}^{2}/\langle \text{Ψ}|{\tilde{U}}^{†}\tilde{U}|\text{Ψ}\rangle$. If and only if Ψ is one of the eigenstates of the NH Hamiltonian with its dissipative nonunitary unit-time evolution of $\tilde{U}$, *P* is maximized to 1. Thus, tuning the angles of the wave plates in state preparation to maximize the measured *P*, the prepared state must tend to one of the target eigenstates (see section S3 and the Supplementary Materials for more details).

### Experimental implementation of $\tilde{U}$

To implement the nonunitary operation $\tilde{U}$, we first decompose it into $\tilde{U}=VD_{3}W$ using singular value decomposition (*47*). The operations of *V* and *W* are unitary and *D*_3_ = ∣*H*_1_〉〈*H*_1_∣ + μ∣*H*_2_〉〈*H*_2_∣ + ν∣*V*_2_〉〈*V*_2_∣ with 0 ≤ μ, ν ≤ 1. We further decompose the two unitary matrices *W* and *V* by the established methods in (*48*, *49*), i.e., *W* = *W*_23_*W*_13_*W*_12_ and *V* = *V*_12_*V*_13_*V*_23_, where *W_ij_* and *V_ij_* are the unitary operations acting on the 2D subspaces of the qutrit system, with the complementary subspace unchanged.

As shown in Fig. 1, the unitary operations of *W_ij_* and *V_ij_* are realized by recombining the photons into the certain spatial mode depending on their polarizations and applying a 2 × 2 transformation via wave plates. The diagonal matrix *D*_3_ can be realized by introducing the mode-selective losses of photons, where the photon losses can be controlled by the HWPs with the setting angles of H_5_ = (arccosν)/2 and H_6_ = (arccosμ)/2.

### Interferometric measurements

As shown in Fig. 1, the single photons are generated by spontaneous parametric down-conversion, and prepared in one of the eigenstates ∣ψ*_j_*〉 of *H*(**k**) with the corresponding eigenenergies of *E_j_*. After photons pass through the NPBS, their state is $(|t\rangle |\psi_{j}\rangle +|r\rangle |\psi_{j}\rangle )/\sqrt{2}$, where *t* and *r* denote the transmitted and reflected modes of the single photons, respectively. Applying the nonunitary operation governed by $\tilde{H}(k)$ on the photons in *t*, the state evolves to $(e^{-i{\tilde{E}}_{j}}|t\rangle |\psi_{j}\rangle +|r\rangle |\psi_{j}\rangle )/\sqrt{2}$. After this stage, the interferometric measurements are performed to obtain the overlap between the states of the photons in the transmitted and reflected modes, and thus to extract the complex phase shift of ${\tilde{E}}_{j}$.

In the measurement stage, an HWP at 45° is first applied on the polarization of the photons in the transmitted mode. After the photons pass through a PBS, their state evolves into

$$\frac{1}{\sqrt{2}}(\alpha_{j}|t_{1}^{\prime }\rangle +\beta_{j}|t_{2}^{\prime }\rangle )(|H\rangle +e^{-i{\tilde{E}}_{j}}|V\rangle )+\frac{1}{\sqrt{2}}\gamma_{j}|r_{2}^{\prime }\rangle (e^{-i{\tilde{E}}_{j}}|H\rangle +|V\rangle )$$

with the assumption ∣ψ_*j*_〉 = α_*j*_∣*H*_1_〉 + β_*j*_∣*H*_2_〉 + γ_*j*_∣*V*_2_〉. Here, $t_{1(2)}^{\prime }$ ($r_{2}^{\prime }$) denotes the transmitted (reflected) mode of the photons that are reflected by the NPBS after passing through the second PBS. Projective measurements are performed on the polarization of the photons in different spatial modes with the bases of $\left\{|\pm \rangle =(|H\rangle \pm |V\rangle )/\sqrt{2},|R\rangle =(|H\rangle -i|V\rangle )/\sqrt{2}\right\}$. The coincidence counts are denoted as $\left\{N_{i}^{\pm },N_{i}^{R}\right\}$, where $i\in \{t_{1}^{\prime },t_{2}^{\prime },r_{2}^{\prime }\}$ corresponds to the spatial mode. Then, we have

$$\xi =\frac{\sum_{i}(N_{i}^{+}-N_{i}^{-})+i\sum_{i}(N_{i}^{+}+N_{i}^{-}-2N_{i}^{R})}{N_{\mathrm{tot}}}-\frac{2i(N_{r_{2}^{\prime }}^{+}+N_{r_{2}^{\prime }}^{-}-2N_{r_{2}^{\prime }}^{R})}{N_{\mathrm{tot}}}$$

where *N*_tot_ denotes the number of the input photons to achieve the state preparation.

Note that our setup can be readily extended to simulate the dynamic evolution and construct the energy spectrum of arbitrary NH Hamiltonians, by taking advantage of the extendable degrees of freedom of photons and specially designed interferometric network (see section S5 and the Supplementary Materials for more details).
