# Supplementary material for: Experimental simulation of symmetry-protected higher-order exceptional points with single photons
Source: Sci Adv. 2023 Aug 23;9(34):eadi0732. doi: 10.1126/sciadv.adi0732 (PMC11801374; doi:10.1126/sciadv.adi0732)
Supplement: Supplementary file 1 — Supplementary Text Figs. S1 to S4 [file sciadv.adi0732_sm.pdf]

Science Advances
AAAS

Supplementary Materials for

Experimental simulation of symmetry-protected higher-order exceptional points with single photons

Kunkun Wang *et al.*

Correspondence author. Wei Yi, wyiz@ustc.edu.cn; Emil J. Bergholtz, emil.bergholtz@fysik.su.se; Peng Xue, gnep.eux@gmail.com

Sci. Adv. **9**, eadi0732 (2023)
DOI: 10.1126/sciadv.adi0732

This PDF file includes:

Supplementary Text
Figs. S1 to S4

S1 PT-symmetry-protected third-order exceptional points.

In our experiment, we first consider the three-band linearized, higher-spin Dirac-like non-Hermitian (NH) Hamiltonians with parity-time (PT) symmetry in two-dimensional (2D) reciprocal space (*35*),

$$H_{PT} = k_x\lambda_1 + i\epsilon(\lambda_2 + \lambda_4 + \lambda_5) + (k_y - i\epsilon)\lambda_6 - (k_y + i\epsilon)\lambda_7 - \frac{\epsilon(\lambda_3 + \sqrt{3}\lambda_8)}{2}. \tag{S1}$$

Here λ_i with $i = 1, 2, \cdots, 8$ denote the Gell-Mann matrices (*44*), i.e.,

$$\lambda_1 = \begin{pmatrix} 0 & 1 & 0 \\ 1 & 0 & 0 \\ 0 & 0 & 0 \end{pmatrix}, \lambda_2 = \begin{pmatrix} 0 & -i & 0 \\ i & 0 & 0 \\ 0 & 0 & 0 \end{pmatrix}, \lambda_3 = \begin{pmatrix} 1 & 0 & 0 \\ 0 & -1 & 0 \\ 0 & 0 & 0 \end{pmatrix}, \lambda_4 = \begin{pmatrix} 0 & 0 & 1 \\ 0 & 0 & 0 \\ 1 & 0 & 0 \end{pmatrix}, \tag{S2}$$

$$\lambda_5 = \begin{pmatrix} 0 & 0 & -i \\ 0 & 0 & 0 \\ i & 0 & 0 \end{pmatrix}, \lambda_6 = \begin{pmatrix} 0 & 0 & 0 \\ 0 & 0 & 1 \\ 0 & 1 & 0 \end{pmatrix}, \lambda_7 = \begin{pmatrix} 0 & 0 & 0 \\ 0 & 0 & -i \\ 0 & i & 0 \end{pmatrix}, \lambda_8 = \begin{pmatrix} \frac{1}{\sqrt{3}} & 0 & 0 \\ 0 & \frac{1}{\sqrt{3}} & 0 \\ 0 & 0 & \frac{-2}{\sqrt{3}} \end{pmatrix}.$$

For the spectrum of H_{PT}, all the three eigenenergies can be uniquely defined as $E_1 = \alpha_+ + \alpha_-$, $E_2 = \omega\alpha_+ + \omega^*\alpha_-$, and $E_3 = \omega^*\alpha_+ + \omega\alpha_-$, where $\omega = (-1 + \sqrt{3}i)/2$ and $\alpha_\pm \equiv \sqrt[3]{q \pm \sqrt{p^3 + q^2}}$ with $p = (-k_x^2 - 2k_y^2 + 4\epsilon^2)/3$ and $q = -\epsilon[k_x^2 - 2k_y^2 + 4(k_y - k_x)\epsilon + \epsilon^2]/2$. The third-order EPs occur when $p = q = 0$ is taken. The exceptional ring (ER) is composed by second-order EPs and exists on the curve of $p^3 + q^2 = 0$, which specifies the PT transitions, with all the eigenvalues being real only in the exact PT region with $p^3 + q^2 > 0$. For $p^3 + q^2 < 0$, PT-symmetry is broken with $E_2 = E_3^*$.

In Figs. 2A and B of the main text, we characterize the energy dispersion along k_y for H_{PT} with $\epsilon = 0$ and $\epsilon = 0.5$. As compensation, we fix $k_y = 0$ with $\epsilon = 0$ and $k_y = -0.5694$ with $\epsilon = 0.5$ for H_{PT} with various k_x experimentally. As shown Fig. S1A, the measured energy spectrum for the Hermitian model with $\epsilon = 0$ presents a triple degeneracy with the same linear dispersion along k_x. The PT-symmetric NH model with $\epsilon = 0.5$ has the energy spectrum which exhibits a cube-root dispersion near the third-order EP along k_x as well [see Fig. S1B].

Additionally, we also study the energy dispersion near the other third-order EP for H_{PT} with $\epsilon = 0.5$. The same features can be derived from the results shown in Figs. S1C and D, where a cube-root energy dispersion away from the third-order EPs located at $(k_x = 0.4701, k_y = 0.6341)$ is observed experimentally.

As shown in Fig. 2E of the main text, the third-order EPs are destroyed by the symmetry-broken perturbation $\sum_{i=1}^{8} \delta_i\lambda_i$ for H_{PT} with $\epsilon = 0.5$. Here δ_i with $i = 1, 2, \cdots, 8$ are chosen randomly in the region of $[-\pi/20, \pi/20]$. In our experiment, we have $\delta_1 = -0.0849$, $\delta_2 = 0.0531$, $\delta_3 = 0.0308$, $\delta_4 = -0.1390$, $\delta_5 = -0.1294$, $\delta_6 = -0.0794$, $\delta_7 = 0.1022$ and $\delta_8 = 0.1114$.

S2 P-symmetry-protected third-order exceptional points.

We consider the P-symmetry-protected NH Lieb lattice model (*35*, *46*),

$$H_P = (1 + \cos k_x - i\epsilon)\lambda_1 + (1 + \cos k_y + i\epsilon)\lambda_6 - \sin k_x \lambda_2 - \sin k_y \lambda_7 \quad \text{(S3)}$$

with eigenenergies 0 and $E_\pm = \pm\sqrt{2(2 + \cos k_x + \cos k_y - i\epsilon \cos k_x + i\epsilon \cos k_y - \epsilon^2)}$. For $\epsilon = 0$, H_P describes the standard nearest neighbor Hermitian model, which possesses a triple energy degeneracy at $k_x = k_y = \pi$. For $\epsilon \neq 0$, the triple energy degeneracy splits into four third-order EPs, which are located at $(k_x = \pm \arccos(\epsilon^2/2 - 1), k_y = \pm \arccos(\epsilon^2/2 - 1))$.

In Figs. 3A and B of the main text, we measure the energy dispersion along k_y by fixing $k_x = \pi$ for H_P with $\epsilon = 0$, and $k_x = 2.6362$ for H_P with $\epsilon = 0.5$. As shown in Figs. S2A and B, by fixing $k_y = \pi$ for H_P with $\epsilon = 0$ and $k_y = 2.6362$ for H_P with $\epsilon = 0.5$, respectively, we sample 11 k_x in our experiment. The measured energy spectrum for H_P with $\epsilon = 0$ presents a triple degeneracy with the linear dispersion along k_x. For H_P with $\epsilon = 0.5$, an anomalous square-root scaling away from the third-order EP along k_x is also observed. In addition, the energy dispersions near the other third-order EP at $(k_x = -2.6362, k_y = -2.6362)$ are shown in Figs. S2C and D. The similar square-root energy dispersion is observed in our experiment.

As shown in Fig. 3D of the main text, the third-order EPs are destroyed by the symmetry-broken perturbation $\sum_{i=1}^{8} \delta_i \lambda_i$ for H_P with $\epsilon = 0.5$. Here δ_i with $i = 1, 2, \cdots, 8$ are chosen randomly in the region of $[-\pi/20, \pi/20]$. In our experiment, we have $\delta_1 = -0.0569$, $\delta_2 = 0.0683$, $\delta_3 = -0.0988$, $\delta_4 = -0.1125$, $\delta_5 = 0.0595$, $\delta_6 = -0.1260$, $\delta_7 = -0.1175$ and $\delta_8 = 0.1544$.

S3 Search for the eigenstates.

As demonstrated in the main text, given an approximate eigenstate, our setup can be used to efficiently estimate the corresponding eigenvalue. However, to calculate the eigenstates by using conventional classical methods requires resources. When the size of the system grows, rapidly increasing of the resources becomes an obstacle. Furthermore, finding the eigenstates of a given Hamiltonian is a fundamental problem and has lots of applications. Here we develop a method of search for the eigenstates of the NH Hamiltonian, which can also be applied to the Hermitian Hamiltonian, apparently.

The search proceeds by preparing the input state of $|\Psi\rangle$. The state is then evolved through a unit-time evolution $\tilde{U} = e^{-i\tilde{H}}$ governed by the NH Hamiltonian $\tilde{H}$. By projecting the evolved state to the initial state, we can obtain the normalized probability of finding the output state unchanged as $P = |\langle\Psi|\tilde{U}|\Psi\rangle|^2/\langle\Psi|\tilde{U}^\dagger\tilde{U}|\Psi\rangle$. For the evolution operator, it can be rewritten as $\tilde{U} = \sum_i e^{-i\tilde{E}_i}|\psi_i\rangle\langle\chi_i|/\langle\chi_i|\psi_i\rangle$. Here $\langle\chi_i|$ and $|\psi_i\rangle$ are the left and right eigenstates of $\tilde{H}$ with the corresponding eigenenergy of $\tilde{E}_i$, which satisfy the relations of

$$\sum_i \frac{|\psi_i\rangle\langle\chi_i|}{\langle\chi_i|\psi_i\rangle} = \mathbb{1},\ \langle\chi_i|\chi_i\rangle = \langle\psi_i|\psi_i\rangle = 1,\ \langle\chi_i|\psi_j\rangle_{i\neq j} = 0.$$

For the input state of $|\Psi\rangle$, it can be represented as

$$|\Psi\rangle = \sum_i \frac{c_i\,|\psi_i\rangle}{\langle\chi_i|\psi_i\rangle} = \sum_i \frac{b_i\,|\chi_i\rangle}{\langle\psi_i|\chi_i\rangle},$$

where $c_i = \langle\chi_i|\Psi\rangle$ and $b_i = \langle\psi_i|\Psi\rangle$. Thus, we have

$$P = \frac{|\langle\Psi|\,\tilde{U}\,|\Psi\rangle|^2}{\langle\Psi|\,\tilde{U}^\dagger\tilde{U}\,|\Psi\rangle} = \sum_{i,j} \frac{c_i^* c_j b_i b_j^* e^{iE_i^*} e^{-iE_j}}{\langle\psi_i|\chi_i\rangle\langle\chi_j|\psi_j\rangle} \Big/ \sum_{i,j} \frac{c_i^* c_j e^{iE_i^*} e^{-iE_j}\langle\psi_i|\psi_j\rangle}{\langle\psi_i|\chi_i\rangle\langle\chi_j|\psi_j\rangle}.$$

Subtracting the numerator from the denominator, we can obtain

$$\begin{aligned}
&\sum_{i,j} \frac{c_i^* c_j e^{iE_i^*} e^{-iE_j}\langle\psi_i|\psi_j\rangle}{\langle\psi_i|\chi_i\rangle\langle\chi_j|\psi_j\rangle} - \sum_{i,j} \frac{c_i^* c_j b_i b_j^* e^{iE_i^*} e^{-iE_j}}{\langle\psi_i|\chi_i\rangle\langle\chi_j|\psi_j\rangle} = \sum_{i,j} \frac{c_i^* c_j e^{iE_i^*} e^{-iE_j}(\langle\psi_i|\psi_j\rangle - b_i b_j^*)}{\langle\psi_i|\chi_i\rangle\langle\chi_j|\psi_j\rangle} \\
&= \sum_{i,j} \frac{c_i^* c_j e^{iE_i^*} e^{-iE_j}\langle\psi_i|(\mathbb{1} - |\Psi\rangle\langle\Psi|)|\psi_j\rangle}{\langle\psi_i|\chi_i\rangle\langle\chi_j|\psi_j\rangle} = \sum_{i,j} \frac{c_i^* c_j e^{iE_i^*} e^{-iE_j} \sum_l \langle\psi_i\,|\Psi_l^\perp\rangle\langle\Psi_l^\perp|\,\psi_j\rangle}{\langle\psi_i|\chi_i\rangle\langle\chi_j|\psi_j\rangle} \\
&= \sum_l |\sum_i \frac{c_i e^{-iE_i}\langle\Psi_l^\perp|\psi_i\rangle}{\langle\chi_i|\psi_i\rangle}|^2 \geq 0,
\end{aligned}$$

where $|\Psi_l^\perp\rangle$ are the states orthogonal to the input state of $|\Psi\rangle$. Thus, we can obtain $P \leq 1$. If and only if $|\Psi\rangle$ is an egigenstate of $\tilde{H}$, the inequality is saturated to an equality with $P = 1$. Therefore, P can act as an eigenstate probe, where it equals to the maximum value of 1 if the input state is an eigenstate of the Hamiltonian.

As proof of principle, we achieve the searching task to prepare the initial state into one of the eigenstates of $\tilde{H}_{PT}$ with $(k_x = -0.6529, k_y = -0.5694)$ and $\epsilon = 0.5$. The setup is shown in Fig. S3A, where the input qutrit state is prepared by tuning the setting angles of the half-wave-plates (HWPs) of H_{1-4}. Then, subjecting the state to the evolution governed by the measured Hamiltonian, the probability P can be measured by subjecting the output state to the reverse process of the initial state preparation. We then have $P = N_1/(N_1 + N_2 + N_3)$, where N_i is the number of heralded clicks at detector D_i. By continuously tuning the angles of H_{1-4} to achieve the maximum P, we can prepare the input state as one of the eigenstate of the Hamiltonian, where the searching task can be interpreted as an optimization problem.

By fixing $(\mathrm{H}_2 = 1.82°, \mathrm{H}_4 = -11.57°)$ and varying the angles of the HWPs $(\mathrm{H}_1, \mathrm{H}_3)$ in state preparation and projective measurement, we obtain the maximum measured $P = 0.982 \pm 0.001$ with $(\mathrm{H}_1 = -6.57°, \mathrm{H}_3 = -5.18°)$. The fidelity between the prepared state and the first eigenstate of $\tilde{H}_{PT}$ is 0.985 ± 0.002.

S4 Generalization to symmetry-protected four-band models.

In our experiment, we first extend the PT-symmetry-protected system to the four-band model governed by the Hamiltonian

$$\begin{aligned} H'_{PT} =& k_x\Gamma_1 + i\epsilon(\Gamma_2+\Gamma_4+\Gamma_5) + (k_y - i\epsilon)\Gamma_6 - (k_y+i\epsilon)\Gamma_7 - \frac{\epsilon}{2}(\Gamma_3+\sqrt{3}\Gamma_8) \\ &+ \frac{\Gamma_0 - \sqrt{6}\Gamma_{15}}{4} \end{aligned} \tag{S4}$$

with the symmetry operators $P' = \mathrm{diag}(1,-1,1,1)$ and $T' = \mathrm{diag}(-1,1,i,1)$. Here Γ_0 is the 4×4 identity matrix and Γ_i ($i = 1, 2, \cdots, 15$) denote the Gell-Mann matrices, that span the Lie algebra of the SU(4) group,

$$\Gamma_1 = \begin{pmatrix} 0&1&0&0\\1&0&0&0\\0&0&0&0\\0&0&0&0 \end{pmatrix}, \Gamma_2 = \begin{pmatrix} 0&-i&0&0\\i&0&0&0\\0&0&0&0\\0&0&0&0 \end{pmatrix}, \Gamma_3 = \begin{pmatrix} 1&0&0&0\\0&-1&0&0\\0&0&0&0\\0&0&0&0 \end{pmatrix}, \Gamma_4 = \begin{pmatrix} 0&0&1&0\\0&0&0&0\\1&0&0&0\\0&0&0&0 \end{pmatrix},$$

$$\Gamma_5 = \begin{pmatrix} 0&0&-i&0\\0&0&0&0\\i&0&0&0\\0&0&0&0 \end{pmatrix}, \Gamma_6 = \begin{pmatrix} 0&0&0&0\\0&0&1&0\\0&1&0&0\\0&0&0&0 \end{pmatrix}, \Gamma_7 = \begin{pmatrix} 0&0&0&0\\0&0&-i&0\\0&i&0&0\\0&0&0&0 \end{pmatrix}, \Gamma_8 = \begin{pmatrix} \frac{1}{\sqrt{3}}&0&0&0\\0&\frac{1}{\sqrt{3}}&0&0\\0&0&\frac{-2}{\sqrt{3}}&0\\0&0&0&0 \end{pmatrix},$$

$$\Gamma_9 = \begin{pmatrix} 0&0&0&1\\0&0&0&0\\0&0&0&0\\1&0&0&0 \end{pmatrix}, \Gamma_{10} = \begin{pmatrix} 0&0&0&-i\\0&0&0&0\\0&0&0&0\\i&0&0&0 \end{pmatrix}, \Gamma_{11} = \begin{pmatrix} 0&0&0&0\\0&0&0&1\\0&0&0&0\\0&1&0&0 \end{pmatrix}, \Gamma_{12} = \begin{pmatrix} 0&0&0&0\\0&0&0&-i\\0&0&0&0\\0&i&0&0 \end{pmatrix},$$

$$\Gamma_{13} = \begin{pmatrix} 0&0&0&0\\0&0&0&0\\0&0&0&1\\0&0&1&0 \end{pmatrix}, \Gamma_{14} = \begin{pmatrix} 0&0&0&0\\0&0&0&0\\0&0&0&-i\\0&0&i&0 \end{pmatrix}, \Gamma_{15} = \begin{pmatrix} \frac{1}{\sqrt{6}}&0&0&0\\0&\frac{1}{\sqrt{6}}&0&0\\0&0&\frac{1}{\sqrt{6}}&0\\0&0&0&-\sqrt{\frac{3}{2}} \end{pmatrix}. \tag{S5}$$

Using the representation of the P-symmetry operator as P', the P-symmetric non-Hermitian Hamiltonian is restricted to the form of

$$H'_P = \frac{(b+d)\Gamma_1 + (f+h)\Gamma_6 + (\beta+\gamma)\Gamma_{11}}{2} + \frac{i\left[(b-d)\Gamma_2 + (f-h)\Gamma_7 + (\beta-\gamma)\Gamma_{12}\right]}{2}, \tag{S6}$$

where $\{b, d, f, h, \beta, \gamma\}$ are arbitrary complex numbers. The specific Hamiltonian satisfies the relations of $\det[H'_P] = \mathrm{Tr}(H'_P) = \mathrm{Tr}(H'^3_P) = 0$ with the corresponding eigenenergies of $\{0, 0, E'_\pm\}$. The two degenerated flat bands with energy zero correspond to the non-defective degeneracies with two distinct eigenstates of $|\psi^0_1\rangle = (-\beta, 0, 0, d)^T/\sqrt{\beta^2+d^2}$ and $|\psi^0_2\rangle =$

$(-f, 0, d, 0)^T/\sqrt{f^2+d^2}$. The two dispersive bands satisfy (*37*)

$$E_\pm = \pm\sqrt{\frac{\mathrm{Tr}(H_P'^2)}{2}} = \pm\sqrt{bd + fh + \beta\gamma}$$

with the corresponding eigenstates of

$$|\psi_3^+\rangle = \frac{(b, \sqrt{bd+fh+\beta\gamma}, h, \gamma)^T}{\mathcal{N}} \text{ and } |\psi_4^-\rangle = \frac{(b, -\sqrt{bd+fh+\beta\gamma}, h, \gamma)^T}{\mathcal{N}},$$

where $\mathcal{N} = \sqrt{b^2+h^2+\gamma^2+bd+fh+\beta\gamma}$. Thus, we tune two parameters to make $bd+fh+\beta\gamma = 0$ satisfied. Then the second-order EPs with $E_\pm = 0$ are generated, which also correspond to the four-fold degeneracy points for the NH system. As shown in Figs. 5C and D of the main text, we choose $b = 1+e^{ik_x} - i\epsilon$, $d = 1+e^{-ik_x} - i\epsilon$, $f = 1+e^{ik_y} + i\epsilon$, $h = 1+e^{-ik_y} + i\epsilon$ and $\beta = \gamma = \sin k_x$. By setting $\epsilon = 0.5$ and tuning k_x and k_y, the two dispersive bands touch each other to form the second-order EPs at $\{\pm 2.7275, \pm 2.7275\}$, where the four-fold degeneracies emerge.

As shown in Fig. S4, to construct the energy spectrum of the symmetry-protected four-band models of H'_{PT} and H'_P in the main text, the basis states are encoded by the hybrid polarization-spatial modes of the single photons as $|0\rangle \Leftrightarrow |H_1\rangle$, $|1\rangle \Leftrightarrow |V_1\rangle$, $|2\rangle \Leftrightarrow |H_2\rangle$, and $|3\rangle \Leftrightarrow |V_2\rangle$. Here $1, 2$ denote the different spatial modes and H (V) denotes the horizontal (vertical) polarization of the single photons. The state preparation is achieved by passing the photons through a polarizing beam splitter (PBS). An adjustable HWP (H_1) combined with a sandwich-type set of HWP (H_2) and quarter-wave plates (QWPs) with the setting angles of 45° are used to control the amplitude and the relative phases of the photon with different polarizations. After passing through a beam displacer (BD), the vertically polarized photons are transmitted and the horizontal polarized photons go through a 3-mm lateral displacement into a neighboring mode. Thus, the photons are prepared into two parallel spatial modes—the transmitted first and lateral second modes. By inserting the wave-plates H_{1-6} into the spatial modes accordingly, we prepare the state of the photons in the eigenstates of the measured Hamiltonian.

After the state preparation, the photons are injected into the $50:50$ non-polarizing beam splitter (NPBS). Thus, extra two paths of the transmitted (t) and reflected (r) modes are introduced. To approach the nonunitary unit-time evolution governed by the mapped NH Hamiltonian on the transmission, we decompose the nonunitary operation as

$$\tilde{U}_4 = e^{-i\tilde{H}'} = V_{34}V_{24}V_{14}V_{23}V_{13}V_{12}D_4W_{12}W_{13}W_{23}W_{14}W_{24}W_{34}.$$

Here $\tilde{H}'$ denotes the mapped NH Hamiltonian of $\tilde{H}'_{PT}$ or $\tilde{H}'_P$. The operations V_{ij} and W_{ij} are the unitary operations acting on the 2D subspaces of the system with the complementary subspace unchanged, and D_4 is a diagonal matrix. As shown in Fig. S4, the unitary operations V_{ij} and W_{ij} can be realized by combining the two acted modes into one spatial mode with different polarizations and applying a 2×2 unitary transformation via the set of wave-plates. The diagonal matrix D_4 can be realized by introducing the mode-selective losses to the corresponding modes, where the intensity of the losses can be controlled by HWPs of H_{7-9}.

After recombining the photons in the t- and r- modes, the projective measurements are performed on the polarization of the photons in different spatial modes with the bases of $\{|\pm\rangle = (|H\rangle \pm |V\rangle)/\sqrt{2}, |R\rangle = (|H\rangle - i|V\rangle)/\sqrt{2}\}$. The coincidences for the projective measurements are counted as $\{N_i^{\pm}, N_i^{R}\}$. Here $i \in \{t'_1, r'_1, t'_2, r'_2\}$ denotes the transmitted (t') or reflected (r') path following the second PBS in the first or second spatial modes of the r-path photons. Accordingly, we can obtain the eigenenergy of $\tilde{H}'$ as the complex phase shift between t and r paths, i.e.,

$$
\begin{aligned}
\xi' = e^{-i\tilde{E}'_j} = \langle\psi_j|\,\tilde{U}\,|\psi_j\rangle = & \frac{\sum_i \left(N_i^+ - N_i^-\right) + i\sum_i \left(N_i^+ + N_i^- - 2N_i^R\right)}{N_{\text{tot}}} \\
& - \frac{2i\left(N_{r'_1}^+ + N_{r'_1}^- - 2N_{r'_1}^R\right) + 2i\left(N_{r'_2}^+ + N_{r'_2}^- - 2N_{r'_2}^R\right)}{N_{\text{tot}}},
\end{aligned}
$$

where N_{tot} denotes the number of the input photons to achieve the state preparation. The eigenenergy E'_j of H'_{PT} or H'_P can thus be obtained through the relation of $E'_j = i\ln\xi' + i\ln\sqrt{\Lambda'}$, where $\Lambda' = \max_{\mathbf{k}}|\zeta_{\mathbf{k}}|$ and $\zeta_{\mathbf{k}}$ is the eigenvalue of $e^{-iH'_{PT}}e^{-iH'^{\dagger}_{PT}}$ or $e^{-iH'_P}e^{-iH'^{\dagger}_P}$.

S5 Generalization to arbitrary models.

Our setup can be efficiently generalized to construct the energy spectrum of arbitrary NH Hamiltonians, by taking advantage of the extendable degrees of freedom of photons and specially designed interferometric network.

As shown in Fig. 1 of the main text, the process starts by preparing the initial state in one of the eigenstates of the corresponding Hamiltonian. For an N-dimensional qudit state, the basis states can be encoded as (*48*)

$$
(|0\rangle, |1\rangle), (|2\rangle, |3\rangle), \cdots, (|N-1\rangle, |N\rangle) \Longleftrightarrow (|H_1\rangle, |V_1\rangle), (|H_2\rangle, |V_2\rangle), \cdots, (|H_n\rangle, |V_n\rangle)
$$

for an even N, where $n = N/2$ denotes the number of the spatial modes and H (V) denotes the horizontal (vertical) polarization of the single photons. Whereas, if N is odd, the basis states are encoded as

$$
(|0\rangle), (|1\rangle, |2\rangle), \cdots, (|N-1\rangle, |N\rangle) \Longleftrightarrow (|H_1\rangle), (|H_2\rangle, |V_2\rangle), \cdots, (|H_n\rangle, |V_n\rangle)
$$

with $n = (N+1)/2$.

To extend the proposed method to achieve the preparation of the initial state, it is convenient to expand the spatial modes by additional BDs and tune the parameters by using the wave-plates. Actually, for an N-dimensional pure states, it can be prepared by using $(N-1)/2$ BDs when N is an odd number and $N/2-1$ for an even number. All the proportions of each mode and the relative phases can be tuned by varying the angles of $N-1$ HWPs and $N-1$ sandwich-type sets of QWP-HWP-QWP.

After passing the initial state through the nonunitary unit-time evolution governed by the mapped NH Hamiltonian, the corresponding eigenenergies will be introduced as the complex phase shift to the evolved state. The decomposition method presented in the main text to achieve the nonunitary dynamics of $\tilde{U}$ can also be generalized to any $N \times N$ passive nonunitary operation as $\tilde{U}_N = \prod_{n=N}^{n=1} \prod_{m=n-1}^{m=1} V_{mn} D_N \prod_{i=1}^{i=N} \prod_{j=i+1}^{j=N} W_{ij}$. Thus, the simple building blocks of BDs and wave plates to approach the corresponding decomposed operations provide a recipe for an implementation of arbitrary $\tilde{U}_N$, experimentally. The maximum numbers of BDs needed to build all the N-dimensional unitary operators of W_{ij} and V_{mn} are both $2N - 4$ when N is an even number and $2N - 3$ for an odd number. The number of BDs to achieve D_N is $N - 1$. Thus, The maximum number of BDs to approach this process is only linear increasing with N. Besides, to fully control the parameters in the nonunitary operation, the maximum number of set of wave plates to approach the decomposed unitary operations is $(N - 1)N$, and $N - 1$ HWPs to realize D_N. At last, by performing interferometric measurements on the photons, the complex eigenenergies for each mode in reciprocal space can be constructed experimentally.

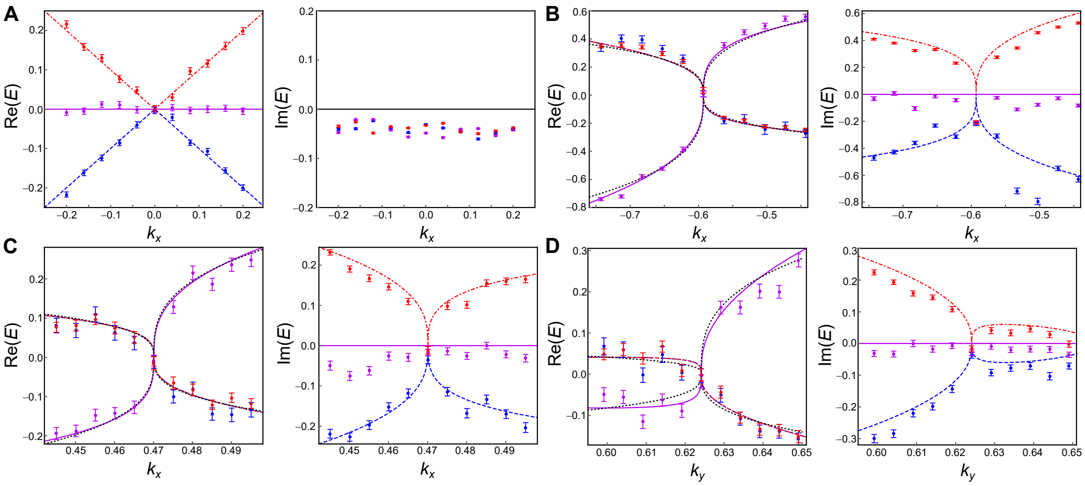

Figure S1: **Energy spectra for the PT-symmetric three-band model.** Measured (dots) and the corresponding theoretical (lines) eigenenergies of the PT-symmetric Hamiltonian H_{PT} with fixed $(k_y = 0, \epsilon = 0)$ for (**A**) and $(k_y = -0.5694, \epsilon = 0.5)$ for (**B**) as a function of the momentum k_x. The energy dispersion near the third-order EP at $(k_x = 0.4701, k_y = 0.6241)$ are observed by fixing $k_y = 0.6241$ along k_x in (**C**), and fixing $k_x = 0.4701$ along k_y in (**D**). The black dotted lines in the left column of (**B**), (**C**) and (**D**) correspond to the results fitted by $\sim k^{1/3}$. Error bars are obtained by assuming Poisson statistics in the photon-number fluctuations, indicating the statistical uncertainty.

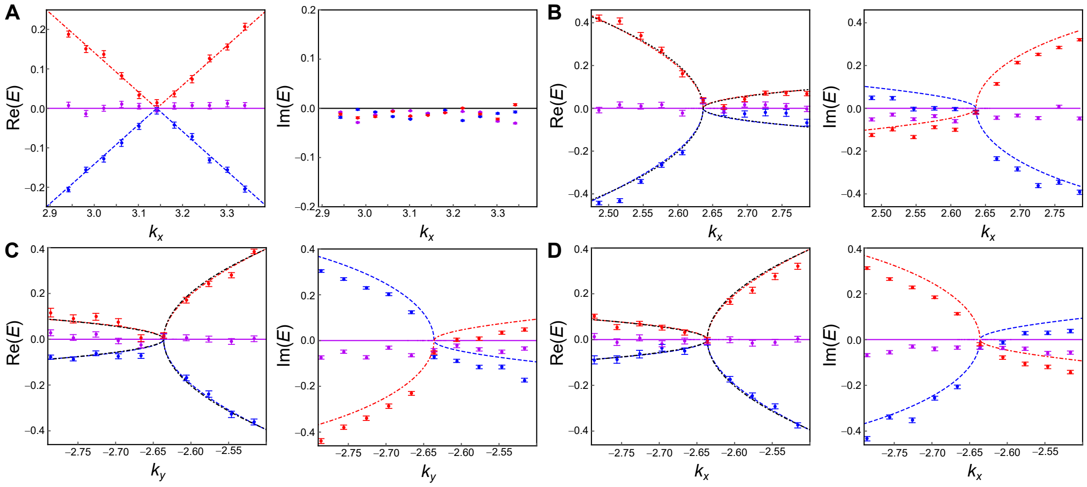

Figure S2: **Energy spectra for the P-symmetric three-band model.** Measured (dots) and the corresponding theoretical (lines) eigenenergies of the P-symmetric Hamiltonian H_P with fixed $(k_y = 0, \epsilon = 0)$ for (**A**) and $(k_y = 2.6362, \epsilon = 0.5)$ for (**B**) as a function of the momentum k_x. The energy dispersion near the other third-order EP at $(k_x = -2.6362, k_y = -2.6362)$ are observed by fixing $k_y = -2.6362$ along k_x in (**C**), and fixing $k_x = -2.6362$ along k_y in (**D**). The black dotted lines in the right column of (**B**), (**C**) and (**D**) correspond to the results fitted by $\sim k^{1/2}$. Error bars are obtained by assuming Poisson statistics in the photon-number fluctuations, indicating the statistical uncertainty.

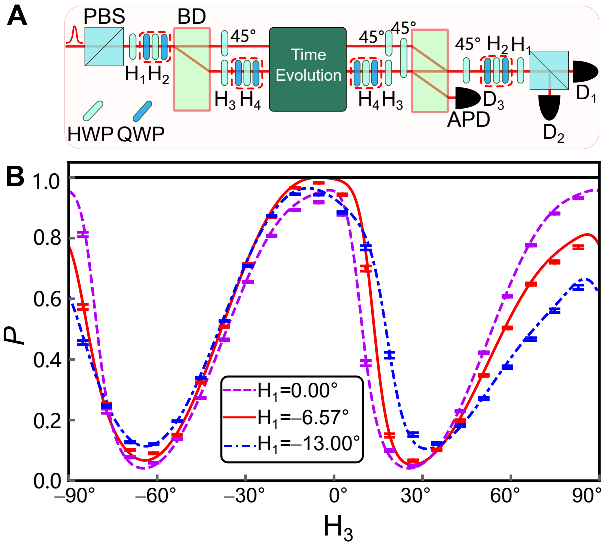

Figure S3: **Search the eigenstates of $\tilde{H}_{PT}$.** (**A**) Schematic of the optical circuit used to search the eigenstates. BD: beam displacer; HWP: half-wave plate; QWP: quarter-wave plate; APD: avalanche photodiode; PBS: polarizing beam splitter. (**B**) The measured results of P by fixing $(\mathrm{H}_2 = 1.82°, \mathrm{H}_4 = -11.57°)$ and varying the angles of the HWPs $(\mathrm{H}_1, \mathrm{H}_3)$. Here the evolution is governed by $\tilde{H}_{PT}$ with $(k_x = -0.6529, k_y = -0.5694)$ and $\epsilon = 0.5$. Theoretical predictions are represented by colored curves, and experimental results by the corresponding symbols. Error bars are obtained by assuming Poisson statistics in the photon-number fluctuations, indicating the statistical uncertainty.

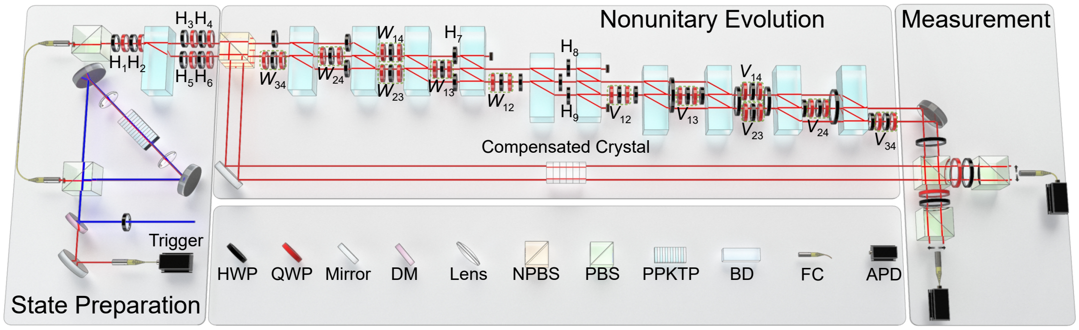

Figure S4: **Experimental setup for the four-band models.** DM: dichroic mirror; NPBS: non-polarizing beam splitter; PPKTP: periodically poled potassium titanyl phosphate crystal; APD: avalanche photodiodes; FC: fiber coupler.
